# Food Processing and Phthalate Exposure: The Nutrition and Health Survey in Taiwan (1993–1996 and 2005–2008)

**DOI:** 10.3389/fnut.2021.766992

**Published:** 2021-11-17

**Authors:** Yi-Chen Huang, Pei-Ru Huang, Yuan-Ting C. Lo, Chien-Wen Sun, Wen-Harn Pan, Shu-Li Wang, Han-Bin Huang

**Affiliations:** ^1^Department of Nutrition, China Medical University, Taichung, Taiwan; ^2^National Defense Medical Center, School of Public Health, Taipei, Taiwan; ^3^National Institute of Environmental Health Sciences, National Health Research Institutes, Miaoli County, Taiwan; ^4^Institute of Biomedical Sciences, Academia Sinica, Taipei, Taiwan

**Keywords:** unprocessed food, ultra-processed food, phthalates, food processing, general population

## Abstract

**Background:** Phthalates esters are widely used commercially and can leach from a food container or food packaging. Few studies have been conducted in Asia regarding food processed to varying levels and human phthalate exposure. This study aimed to evaluate the association between unprocessed and ultra-processed food intake and urinary phthalate metabolite levels in the Taiwanese adult population.

**Methods:** A total of 516 participant data were extracted from the cross-sectional 1993–1996 and 2005–2008 Nutrition and Health Survey in Taiwan of those aged over 18 years, where urinary measures and one 24-h dietary recall were collected. Urinary concentrations of dimethyl phthalate, diethyl phthalate, dibutyl phthalate, butyl benzyl phthalate, and di-(2-ethylhexyl) phthalate metabolites including monomethyl phthalate, monoethyl phthalate (MEP), monobutyl phthalate (MBP), monobenzyl phthalate, mono-(2-ethylhexyl) phthalate, mono-(2-ethyl-5-hydroxyhexyl) phthalate, and mono-(2-ethyl-5-oxohexyl) phthalate were measured in spot urine samples. The NOVA food processing classification system was applied to divide all consumed foods into four mutually exclusive groups including unprocessed or minimally processed, processed culinary ingredients, processed and ultra-processed food. Generalized linear models were employed to examine the associations between the percentage quartiles (Qs) of unprocessed and ultra-processed foods in the total weight of food and the urinary phthalate metabolites.

**Results:** Compared with participants in the lowest quartiles (Q1) of ultra-processed food intake, highest ultra-processed food intake (Q4) had 65.7% (95% confidence interval [CI]: 4.83, 162) higher urinary concentrations of MEP after adjusted for covariates. In contrast, the higher unprocessed food consumption was inversely associated with urinary concentrations of MEP and MBP (*P* for trend = 0.03). When compared to the lowest unprocessed food consumers (Q1), higher consumers (Q4) presented 38.6% (95% CI: −61.3, −2.59) lower MEP concentrations and 23.1% (95% CI: −38.5, −3.71) lower MBP concentrations.

**Conclusion:** Ultra-processed food consumption was associated with increased concentrations of urinary MEP. Conversely, consuming unprocessed food was associated with lower concentrations of MEP and MBP in the Asian Taiwanese adult population.

## Introduction

NOVA classification system defines ultra-processed food as industrial formulations manufactured from substances derived from whole foods and that typically contain cosmetic additives such as added flavors and colors (1). Most ultra-processed foods, such as ready-to-eat meals, sugar-sweetened beverages, and packaged snack products, contain little or no nutrients but high levels of calories, fat, sugar, and additives. Ultra-processed food consumption has been associated with an increased risk of obesity (2), type 2 diabetes (3), cardiovascular disease (4), cancer (5), and premature death (6). Thus, this is a concerning health issue globally.

Phthalate esters (PAEs) are a class of high production volume industrial chemicals and are widely used commercially (7). High-molecular-weight (HMW) phthalates, such as di-(2-ethylhexyl) phthalate (DEHP), are mainly used for food containers, food packaging, and the polyvinyl chloride (PVC) gloves worn for food preparation (8). Food intake constitutes 60 to 80% of DEHP and di-n-butyl phthalate (DnBP) exposure (9). Low-molecular-weight (LMW) phthalates, such as dimethyl phthalate (DMP) and diethyl phthalate (DEP), are commonly used in personal care products, or in the printing ink on food packaging (8). DEHP, diisobutyl phthalate (DiBP) and DnBP were the most detected phthalate compound in 400 different food and packaging samples on the Belgian market by a national survey (10). In Taiwan, the highest concentrations of di-n-butyl phthalate (DBP) and DEHP were found in dairy products, vegetable oil, tea drinks and carbonated drinks. Furthermore, the polyvinylidene chloride (PVDC) and polypropylene (PP) used in the food package materials or containers had the highest released of DBP and DEHP, respectively (11). Thus, phthalate contamination may be a result of the use of PVC gloves for food preparation or phthalate migration from food containers or packaging. Phthalate esters are endocrine-disrupting chemicals that can lead to increase the risk of reproductive problems (12), insulin resistance, and metabolic syndrome (7).

Phthalates can leach, migrate, or off-gas from plastic products in the high temperature environment, entering the body by ingestion, inhalation, or dermal absorption (13). Once absorbed, phthalates are rapidly metabolized by hydrolysis and subsequent oxidation reactions and excreted in urine, with an elimination half-life of 24–48 h (14). Human biomonitoring studies have indicated that the simple monoesters are the major urinary metabolites of short-chain phthalates such as DnBP, DMP and DEP (15). In the case of long-chain phthalates such as DEHP, most of the simple monoester such as mono-(2-ethylhexyl) phthalate (MEHP) is further metabolized to a number of oxidative metabolites. The secondary oxidized metabolites such as mono-(2-ethyl-5-hydroxyhexyl) phthalate (MEHHP) and mono-(2-ethyl-5-oxohexyl) phthalate (MEOHP) are the main metabolites excreted in human urine (16). Urinary phthalate metabolites are therefore regarded as biomarkers of phthalate exposure in people of all ages (14, 17–19).

Diet is one major source or pathway for phthalate exposure. Food monitoring studies have demonstrated that higher urinary concentrations of several phthalate metabolites (HMWs and LMWs) were positively associated with meat, dairy, and seafood products and fast food consumption but negatively associated with fruit and vegetable consumption (20–22). Several hypotheses were proposed to explain how substances are created during food processing and how contaminants migrate from packaging to food (23). A few epidemiological studies have observed that unprocessed and ultra-processed food intake were associated with urinary levels of some phthalate metabolites in United States (24, 25). However, the association has yet to be determined in the Asia population with a different dietary culture, where ultra-processed food sale and consumption has increased along with economic growth rates and growing populations (26, 27). Hence, this study investigated the association between the proportion of unprocessed and ultra-processed food intake as part of total food intake and urinary phthalate metabolite concentrations in the Taiwanese adult population.

## Materials and Methods

### Participants

All participants were enrolled from the Nutrition and Health Survey in Taiwan (NAHSIT) conducted in 1993–1996 (NAHSIT I) (28) and 2005–2008 (NAHSIT II) (29). The details of the study design have been published elsewhere (30, 31). A total of 1,109 participants (NAHSIT I: 591; II: 518) were selected to examine urine phthalate exposure on the basis of the probability proportional to size according to regional strata. We then excluded 593 enrollees because they were under 18 years old (*n* = 47), had no urinary specimen (*n* = 234) or dietary information (*n* = 239), or had atypical total daily energy intake (male: <800 kcal/d or >4,000 kcal/d; female: <500 kcal/d or >3,500 kcal/d; *n* = 73) (32). Finally, 516 participants with complete dietary and phthalate metabolite data were enrolled for data analysis. The study protocol was approved by the Research Ethics Committee of China Medical University and Hospital (No: CMUH108-REC2-174). All participants provided informed consent.

### Food Processing Classification

Trained interviewers collected demographic information and information on personal behavior, disease history, and diet through face-to-face interviews. The 24-h dietary recall approach was employed to record all food and beverages consumed during the 24 h prior to interview. The interviewer used food models, measuring cups, spoons, electronic scales, and cue cards to ensure the accuracy of participants' estimations of the weight of their food intake (33).

We employed the NOVA food processing classification system developed by Monteiro et al. (1, 34) to define the level of food processing of each food and beverage consumed within the 24-h period. Composite dishes and baked goods were unfolded into underlying ingredients/food if homemade. Then, all individual food items were classified into the unprocessed group (e.g., peanut), processed culinary ingredients group (e.g., peanut oil), processed food group (e.g., canned peanuts), or ultra-processed food (e.g., peanut-flavored soy milk) according to the definition of NOVA classification system. The definition and examples of unprocessed and ultra-processed foods can be found the below and in Supplementary Table S1.

1. Unprocessed or minimally processed foods: Unprocessed foods are largely of plant or animal origin. Minimal processing is employed to preserve and prolong shelf life or enhance nutrition quality, and cosmetic food additives are not used (e.g., frozen vegetables, rice, milk, plain yogurt, tea). We divided these foods into the following nine subgroups: meat and viscera, fruit and 100% fruit juice, milk and plain yogurt, whole grains, eggs, legumes, fish and seafood, vegetables, and other foods such as nuts.
2. Processed culinary ingredients: Ingredients are extracted and purified from constituents of foods or from nature and used as seasoning in the preparation and cooking of meals (e.g., salt, sugar, oil).
3. Processed foods: Industrial products made by adding processed culinary ingredients to group 1 foods to enhance durability, palatability, and appeal. Preservation methods such as canning, bottling, salting, smoking, and curing are used (e.g., fish preserved in oil, canned nuts, ham, bacon, tofu, cheese).
4. Ultra-processed foods: Ready-to-eat/heat industrial formulations of food ingredients that have been derived from whole foods, and that typically contain cosmetic additives (such as emulsifiers, sweeteners, colors, and flavors). We divided these foods into the following 11 subgroups based on ingredients and characteristics (24, 27, 34): breads, drinks, cookies, frozen and ready-to-eat products, grain products, sauces and dressings, reconstituted meat, milk-based products (e.g., ice cream), sweet snacks, fried foods, and others such as vegetarian meat substitutes.

We summed the weight of each food (g/d) consumed by each participant across the four NOVA food groups. Then, we calculated the percentage (%) of the total food weight (including beverages) of the unprocessed and ultra-processed foods. This approach (instead of energy contribution) was also employed in previous studies in order to account for the effects of food that provided no energy (e.g., artificially sweetened beverages) or nutritional elements (e.g., food additives) in relation to food processing (4, 5).

### Measurement of Urinary Phthalate Metabolite Levels

Spot urine samples collected in the early morning of the health examination from each participant were examined for concentrations of seven phthalate metabolites, monomethyl phthalate (MMP), monoethyl phthalate (MEP), monobutyl phthalate (MBP), monobenzyl phthalate (MBzP), mono-(2-ethylhexyl) phthalate (MEHP), mono-(2-ethyl-5-hydroxyhexyl) phthalate (MEHHP), and mono-(2-ethyl-5-oxohexyl) phthalate (MEOHP). We quantified seven phthalate metabolite levels using quantitative liquid chromatography-electrospray ionization tandem mass spectrometry, as described elsewhere (35). The sum of the DEHP metabolites (ΣDEHP) was calculated by adding the molecular-based concentrations of MEHP, MEHHP, and MEOHP. The limit of detection (LOD) of MMP, MEP, MBP, MBzP, MEHP, MEHHP, and MEOHP was 0.3, 0.3, 1, 0.3, 0.7, 0.1, and 0.1 ng/mL^−1^, respectively. When the phthalate metabolite levels were below the LOD, we replaced these values with half of the LOD (36). The percentage of urinary phthalate metabolites higher than their LOD ranged from 88 to 100%. A blank repeated quality control (QC) sample was included in each batch of the urinary samples, with QC sample concentrations < two-fold the method detection limit. The intraday variations were <10%, and the intraday recoveries were 100 ± 20% at three different concentrations, 25, 50, and 75%, of the individual substances.

### Covariates

We collected demographics, urinary creatinine levels, and anthropometrics at baseline through a questionnaire and health examination. We divided age into four groups ( ≤ 25, 26–45, 46–65, and >65), gender into two groups (male, female), and education level into four groups (illiterate/elementary school, junior high school, senior high school, and college or above). We calculated body mass indices (BMIs) by weight (kg)/height (m)^2^ and divided them into four groups (underweight: <18.5; normal weight: 18.5–23.9; overweight: 24–26.9; obese: ≥27 kg/m^2^) using the Health Promotion Administration, Ministry of Health and Welfare of Taiwan (2018) classifications for adults (37). The frequency, type, and duration of physical activity was collected using the International Physical Activity Questionnaire. The type and duration of physical activity in the previous week were converted to the metabolic equivalent of task (MET-h) per week for each individual. Levels of physical activity were classified into three groups (low: <10; moderate: 10–50; high: ≥50 MET-h/week) according to the MET (38, 39). Having chronic kidney disease and habits of smoking or drinking were defined by participant's self-report. The metabolic syndrome was evaluated by the United States National Cholesterol Education Program/Adult Treatment Panel III definition (40) with modified central obesity and fasting glucose criteria (41). We calculated total energy and nutrient intake for every food and beverage recorded in the 24-h dietary recall. The nutrient content of each food was measured in reference to the Taiwan Food Composition Table (33, 42). Nutrient intake was presented as nutrient density (per 1,000 kcal). The survey period was defined by the NAHSIT I and II to control time effects.

### Statistical Analysis

All data were analyzed using SAS version 9.4 (SAS Institute., Cary, NC, USA). We transformed the phthalate metabolites with the base-10 logarithm to fit the normal distribution. Phthalate metabolites were presented as geometric means (95% confidence interval [CI]). We used analysis of variance to examine the difference between phthalate metabolites on the basis of the percentage quartiles (Qs) of unprocessed and ultra-processed foods in the total food and beverage intake weight. The Bonferroni correction was used as the *post-hoc* test.

Generalized linear models were employed to examine the association between the percentage quartiles of food weight of unprocessed and ultra-processed foods and the log-transformed phthalate metabolites; 100 × [10^(β)^ – 1] was used to calculate the percentage difference of phthalate metabolites between quartiles for unprocessed and ultra-processed foods. The model was adjusted for survey year (NAHSIT I and II), age ( ≤ 25, 26–45, 46–65, and >65), sex (male/female), BMI (<18.5, 18.5–23.9, 24–26.9, and ≥27 kg/m^2^), education level (illiterate or elementary school, junior high school, senior high school, and college or above), total energy intake, and urinary creatinine value. These covariates in the models were selected on the basis of a previous study (24) and a 10% change-in-estimation criterion (43). Tests of linear trend were performed in order to evaluate the effect of quartiles of the percentages of unprocessed or ultra-processed foods as single continuous variables. Sensitivity analysis further adjusted for total fat intake.

## Results

### Baseline Characteristics by Level of Food Processing

A total of 71.3 and 56.3% of food weight and calories were contributed by unprocessed/minimally processed foods, respectively. In contrast, 16.3 and 18.8% of food weight and calories were contributed by ultra-processed foods (Supplementary Table S2). Baseline characteristics of study participants by quartiles of unprocessed and ultra-processed food intake are detailed in Table 1. Those with the highest unprocessed food intake (Q4) tended to be older (>65 years, 17.1 vs. 1.55%), had a lower education level (below elementary school, 41 vs. 11%) and total energy intake (1,545 vs. 1,886 kcal), and higher protein (41.9 vs. 37.0 g/1,000 kcal), dietary fiber (11.1 vs. 6.15 g/1,000 kcal), calcium (275 vs. 195 mg/1,000 kcal) and magnesium (140 vs. 112 mg/1,000 kcal) intake compared with the lowest group (Q1; all *P* < 0.05). Compared with the lowest ultra-processed intake group (Q1), participants with the highest ultra-processed intake (Q4) were younger ( ≤ 25 years, 31 vs. 11.6%; in the 2005–2008 survey period, 72.9 vs. 47.3%), had a higher education level (college or above, 33.6 vs. 11.3%), total energy intake (1,894 vs. 1,500 kcal), and fat intake (34.4 vs. 28.3 g/1,000 kcal) but a lower protein (36.8 vs. 42.4 g/1,000 kcal), dietary fiber (6.35 vs. 10.8 g/1,000 kcal), calcium (205 vs. 226 mg/1,000 kcal) and magnesium (111 vs. 131 mg/1,000 kcal) intake (*P* values were all significant).

**Table 1:** Baseline characteristics of study participants by quartiles of unprocessed and ultra-processed food intake.

**Variables**	**Total**	**Unprocessed food**		***P* value**	**Ultra-processed food**		***P* value**
	**(*n* = 516)**	**Quartile 1**	**Quartile 4**		**Quartile 1**	**Quartile 4**	
		** <56.9%**	**>88.45%**		** <0.89%**	**>26.1%**	
n		129	129		129	129	
Age (years)				<0.0001			<0.0001
≤ 25	85 (16.5)	35 (27.1)	11 (8.53)		15 (11.6)	40 (31.0)	
26–45	237 (45.9)	75 (58.1)	41 (31.8)		54 (41.9)	71 (55.0)	
46–65	153 (29.7)	17 (13.2)	55 (42.6)		50 (38.8)	15 (11.6)	
>65	41 (7.95)	2 (1.55)	22 (17.1)		10 (7.75)	41 (7.95)	
Gender – male	294 (48.1)	66 (51.2)	61 (47.3)	0.09	72 (47.4)	83 (54.3)	0.50
Survey year				0.19			0.0003
1993–1996	197 (38.2)	39 (30.2)	51 (39.5)		68 (52.7)	35 (27.1)	
2005–2008	319 (61.8)	90 (69.8)	78 (60.5)		61 (47.3)	94 (72.9)	
Education level				<0.0001			<0.0001
Illiterate/elementary school	125 (25.2)	14 (11.0)	48 (41.0)		43 (34.7)	13 (10.2)	
Junior high school	91 (18.4)	20 (15.6)	21 (18.0)		30 (24.2)	19 (14.8)	
Senior high school	175 (35.3)	53 (41.4)	35 (29.9)		37 (29.8)	53 (41.4)	
College or above	105 (21.2)	41 (32.0)	13 (11.1)		14 (11.3)	43 (33.6)	
Body mass index (kg/m^2^)				0.24			0.29
Underweight (<18.5)	48 (9.30)	15 (11.6)	8 (6.20)		10 (7.75)	13 (10.1)	
Normal weight (18.5–23.9)	237 (45.9)	66 (51.2)	63 (48.8)		58 (45.0)	67 (51.9)	
Overweight (24.0–26.9)	124 (24.0)	25 (19.4)	27 (20.9)		32 (24.8)	26 (20.2)	
Obesity (≥27)	107 (20.7)	23 (17.8)	31 (24.0)		29 (22.5)	23 (17.8)	
Physical activity (MET, h/week)				0.28			0.35
Low (<10)	144 (37.2)	28 (33.3)	34 (32.4)		43 (40.2)	26 (31.0)	
Moderate (≥10– <50)	169 (43.7)	41 (48.8)	44 (41.9)		38 (35.5)	42 (50.0)	
High (≥50)	74 (19.1)	15 (17.9)	27 (25.7)		26 (24.3)	16 (19.1)	
Current smoker	174 (35.2)	49 (39.2)	41 (33.6)	0.76	47 (38.5)	43 (34.1)	0.85
Current drinker	255 (51.7)	65 (52.4)	53 (43.4)	0.10	62 (50.8)	61 (48.8)	0.84
Disease history							
Kidney disease	13 (2.59)	5 (3.97)	2 (1.59)	0.25	2 (1.59)	3 (2.36)	0.82
Metabolic syndrome	88 (18.5)	17 (14.7)	21 (17.7)	0.55	21 (16.9)	15 (13.2)	0.11
Nutrients intake							
Total energy intake (kcal/d)	1714 (958)	1886 (1037)	1545 (812)	<0.0001	1500 (800)	1894 (1033)	<0.0001
Carbohydrate (g/1,000 kcal)	134 (38.1)	134 (39.1)	138 (44.7)	0.76	138 (47.8)	133 (40.4)	0.39
Fat (g/1,000 kcal)	31.9 (15.3)	33.7 (15.7)	28.9 (18.4)	0.27	28.3 (19.0)	34.4 (15.8)	0.03
Protein (g/1,000 kcal)	40.6 (15.4)	37.0 (14.1)	41.9 (14.3)	0.001	42.4 (17.5)	36.8 (15.0)	0.008
Dietary fiber (g/1,000 kcal)	8.96 (6.86)	6.15 (4.35)	11.1 (7.01)	<0.0001	10.8 (6.61)	6.35 (3.77)	<0.0001
Calcium (mg/1,000 kcal)	244 (258)	195 (228)	275 (317)	0.006	226 (227)	205 (210)	<0.0001
Magnesium (mg/1,000 kcal)	129 (71.8)	112 (55.9)	140 (83.4)	<0.0001	131 (75.2)	111 (50.8)	<0.0001

*Data presented as n (%) or median (interquartile range)*.

*P values were tested using chi-square and Kruskal–Wallis tests for categorical variables and continuous variables, respectively*.

### Distribution of Urinary Phthalate Metabolites by Level of Food Processing

The geometric means (95% CI) of urinary phthalate metabolites for unprocessed and ultra-processed food groups are presented in Table 2. We recorded the lowest concentrations of phthalate metabolites for the unprocessed food intake group in Q4; these participants exhibited significantly lower concentrations of MEP (11.6 vs. 26.8 μg/L), MBP (35.5 vs. 59.3 μg/L), and DEHP (0.21 vs. 0.31 μmol/L) compared with those in Q1 (all *P* < 0.01). Compared with Q1, Q3 and Q4 of the ultra-processed food intake group exhibited significantly higher MEP (25.8 μg/L in Q3 and 28.3 μg/L in Q4 vs. 12.4 in Q1 μg/L). For MBP, participants in Q3 of the ultra-processed food intake group had significantly higher concentrations compared with Q1 (57.8 vs. 40.6 μg/L; *P* < 0.01). Participants in Q3 and Q4 of the ultra-processed food intake group exhibited higher urinary concentrations of MMP, MBzP, and DEHP; however, they were not significant.

**Table 2:** Geometric means (95% confidence interval) of urinary phthalate metabolites by quartiles of unprocessed and ultra-processed food intake (*n* = 516).

**Variables**	**Quartiles**				***P* value**
	**1 (Low)**	**2**	**3**	**4 (High)**	
**Unprocessed food**	<56.9%	56.9–76.6%	76.7–88.4%	>88.4%	
MMP (μg/L)	8.32 (6.48, 10.7)	8.89 (6.86, 11.5)	7.82 (5.81, 10.5)	6.98 (5.74, 8.91)	0.62
MEP (μg/L)	26.8 (18.9, 37.8)^a^	26.2 (19.8, 34.8)^b^	19.0 (13.8, 26.0)	11.6 (8.46, 15.8)^ab^	0.0005
MBP (μg/L)	59.3 (49.3, 71.3)^a^	53.5 (44.8, 63.9)	46.8 (39.1, 56.0)	35.5 (29.8, 45.3)^a^	0.0005
MBzP (μg/L)	1.94 (1.53, 2.46)	1.81 (1.42, 2.31)	1.88 (1.47, 2.39)	1.61 (1.27, 2.04)	0.72
ΣDEHP (μmol/L)	0.31 (0.25, 0.37)	0.33 (0.27, 0.39)^a^	0.31 (0.25, 0.38)	0.21 (0.18, 0.25)^a^	0.004
**Ultra-processed food**	<0.89%	0.89–9.38%	9.39–26.1%	>26.1%	
MMP (μg/L)	7.81 (6.18, 9.87)	7.58 (5.72, 10.0)	8.22 (6.26, 10.8)	8.29 (6.36, 10.8)	0.96
MEP (μg/L)	12.4 (9.04, 17.0)^ab^	17.1 (12.6, 23.1)	25.8 (18.7, 35.6)^a^	28.3 (20.5, 39.0)^b^	0.0007
MBP (μg/L)	40.6 (33.9, 48.7)	41.3 (34.4, 49.6)	57.8 (47.9, 69.7)	54.3 (45.8, 64.3)	0.008
MBzP (μg/L)	1.65 (1.30, 2.11)	1.58 (1.24, 2.02)	2.12 (1.67, 2.71)	1.91 (1.53, 2.40)	0.29
ΣDEHP (μmol/L)	0.27 (0.22, 0.33)	0.26 (0.22, 0.32)	0.30 (0.26, 0.35)	0.31 (0.26, 0.37)	0.53

*ANOVA was used to evaluate the different between phthalate metabolites and groups of unprocessed or ultra-processed food. The same superscript indicated statistically significant differences between two groups using a Bonferroni correction—significance level of P < 0.0083 (0.05/6)*.

*Data presented as geometric means (95% confidence interval)*.

### Association Between Level of Food Processing and Urinary Phthalate Metabolites

Table 3 presents the percentage difference (95% CI) in urinary phthalate metabolites among the quartiles of unprocessed and ultra-processed food intake. The percentages (95% CI) of MEP concentrations for Q2, Q3, and Q4 in unprocessed food intake were −1.92 (−36.8, 52.3), −29.1 (−54.3, 10.3), and −56.8 (−72.2, −32.8), respectively, compared with Q1 in the crude model (*P* for trend <0.01). After adjustment for confounders, the percentage difference in Q4 was −38.6 (−61.3, −2.59; *P* for trend = 0.03). The same pattern was recorded for MBPs. Participants with unprocessed food intake in Q4 had a 40.1% lower concentration of MBPs (95% CI: −53.4, −23.1) compared with Q1. The percentage difference was −23.1 (95% CI: −38.5, −3.71) after adjustment for confounders (*P* for trend = 0.03). For DEHP concentration, Q4 of unprocessed food intake exhibited a 30.5% lower rate (95% CI: −46.2, −10.2) compared with Q1 (*P* for trend = 0.007). However, this was non-significant after adjustment for other confounders (−16.5, 95% CI: −35.5, 8.17).

**Table 3:** Percentage difference (95% confidence interval) in urinary phthalate metabolites (μg/L) by quartiles of unprocessed and ultra-processed food intake (*n* = 516).

**Variables**			**Quartile**				***P* for trend**
			**1 (Low)**	**2**	**3**	**4 (High)**	
**Unprocessed food**							
LMWP	MMP	Crude model	Ref.	6.78 (−26.0, 54.2)	−6.07 (−35.0, −35.6)	−16.1 (−41.9, 21.1)	0.27
		Adjusted model	Ref.	3.32 (−26.1, 44.4)	−3.42 (−31.7, 36.5)	0.02 (−30.6, 44.1)	0.91
	MEP	Crude model	Ref.	−1.92 (−36.8, 52.3)	−29.1 (−54.3, 10.3)	**−56.8 (−72.2**, **−32.8)**	<0.0001
		Adjusted model	Ref.	10.3 (−27.7, 68.3)	−9.76 (−41.7, 39.7)	**−38.6 (−61.3**, **−2.59)**	0.03
	MBP	Crude model	Ref.	−9.74 (−29.7, 15.9)	−21.1 (−38.5, 1.34)	**−40.1 (−53.4**, **−23.1)**	<0.0001
		Adjusted model	Ref.	−9.20 (−26.1, 11.6)	−10.3 (−27.5, 11.0)	**−23.1 (−38.5**, **−3.71)**	0.03
HMWP	MBzP	Crude model	Ref.	−6.57 (−33.2, 30.6)	−3.13 (−30.7, 35.4)	−16.9 (−40.5, 16.2)	0.34
		Adjusted model	Ref.	−2.73 (−28.7, 32.7)	8.72 (−21.1, 49.8)	8.47 (−22.6, 52.1)	0.52
	ΣDEHP	Crude model	Ref.	6.64 (−17.5, 37.8)	1.86 (−21.2, 31.7)	**−30.5 (−46.2**, **−10.2)**	0.007
	(μmol/L)	Adjusted model	Ref.	6.12 (−16.3, 34.5)	13.0 (−11.6, 44.4)	−16.5 (−35.5, 8.17)	0.28
**Ultra-processed food**							
LMWP	MMP	Crude model	Ref.	−2.95 (−32.8, 40.2)	5.32 (−27.1, 52.2)	6.12 (−26.6, 53.4)	0.66
		Adjusted model	Ref.	6.71 (−24.1, 50.0)	0.18 (−29.1, 41.5)	3.66 (−27.8, 48.9)	0.93
	MEP	Crude model	Ref.	37.9 (−11.2, 114)	**108 (33.9, 223)**	**128 (47.0, 255)**	<0.0001
		Adjusted model	Ref.	53.1 (−0.44, 135)	**75.1 (13.2, 171)**	**65.7 (4.83, 162)**	0.03
	MBP	Crude model	Ref.	1.62 (−21.0, 30.7)	**42.2 (10.6, 82.8)**	**33.6 (3.94, 71.8)**	0.003
		Adjusted model	Ref.	12.2 (−9.07, 38.4)	22.2 (−1.19, 51.3)	10.6 (−11.5, 38.3)	0.28
HMWP	MBzP	Crude model	Ref.	−4.46 (−31.6, 33.4)	28.4 (−8.08, 79.3)	15.7 (−17.2, 61.6)	0.18
		Adjusted model	Ref.	0.00 (−27.1, 37.1)	13.2 (−17.8, 57.8)	−2.77 (−30.5, 36.0)	0.94
	ΣDEHP	Crude model	Ref.	−2.32 (−24.7, 26.6)	11.6 (−13.9, 44.6)	15.2 (−11.1, 49.4)	0.18
	(μmol/L)	Adjusted model	Ref.	4.59 (−18.0, −33.3)	0.23 (−21.8, 27.9)	−0.18 (−22.9, 29.2)	0.92

*LMWP, low-molecular-weight phthalates; HMWP, high-molecular-weight phthalates*.

*Model was adjusted for survey year, age, gender, body mass index, education level, total energy intake, and urinary creatinine*.

*P < 0.05 is presented in bold*.

For ultra-processed food intake, those in Q3 and Q4 exhibited a 108% (95% CI: 33.9, 223) and 128% (95% CI: 47.0, 255) increase in MEP concentration compared with Q1, respectively (*P* for trend < 0.001). After adjustment for confounders, the percentage differences were 75.1 (13.2, 171) and 65.7 (4.83, 162), with *P* = 0.03 for the trend. For MBP, those of ultra-processed intake in Q3 and Q4 exhibited 42.2% (10.6, 82.8%) and 33.6% (3.94, 71.8) increased concentrations, respectively (*P* for trend < 0.01). However, this was non-significant after adjustment for covariates.

No substantial differences in association nor statistical significance were observed after further adjustment for total fat intake compared with the original findings (Supplementary Table S3).

## Discussion

In cross-sectional surveys conducted from 1993 to 1996 and from 2005 to 2008, the high ultra-processed food intake of Taiwanese participants was significantly correlated with high concentrations of MEP urinary phthalate metabolites. Participants with high ultra-processed intake (Q4) had 65.7% higher concentrations of MEP than those with a low intake (Q1). High unprocessed food consumption was inversely associated with urinary concentrations of MEP and MBP. Participants with the highest quartiles of unprocessed food intake as a proportion of their total food weight intake exhibited 23.1 and 38.6% lower MBP and MEP concentrations, respectively, compared with the lowest group. These results may indicate that ultra-processed food consumption is a contributor of phthalate exposure in the study population.

Buckley et al. used the US National Health and Nutrition Examination Survey (NHANES) 2013–2014 to assess the association between ultra-processed food consumption and exposure to phthalates; the results revealed that the high energy from ultra-processed food was associated with high urinary concentrations of MCPP, MCNP, and MCOP (24). In a cross-sectional study of the US population aged 6 years and above using the 2009–2016 NHANES, Martínez Steele et al. provided evidence for high ultra-processed food consumption exhibiting a positive dose–response association with urinary concentrations of ΣDiNP, MCNP, MCPP, and MBzP (25). Furthermore, Zota et al. observed a positive dose–response association between high fast food consumption and high urinary levels of ΣDEHP metabolites in multiple NHANES survey cycles (2003–2004, 2005–2006, 2007, 2008, 2009–2010) (13). Our results demonstrated that the higher the ultra-processed food intake is, the higher the concentrations of urinary MEP are in adults. Discrepancies with the aforementioned studies could be attributed to differences in age, exposure profiles, sampling periods, race, food classifications, regions, and sample sizes. Before 2011, the Taiwan Food and Drug Administration (TFDA) did not regulate the passing standard of phthalates [e.g., DEHP, DBP, benzyl butyl phthalate (BBP), DMP and DEP] in food utensils, containers, and packages (Sanitation Standard for Food Utensils, Containers and Packages, DOH Food No.0991303265, 11/22/2010). In the Sanitation Standard and Article 6, only Pb and Cd in the plastics shall meet the requirements for food utensils, containers and packages (44). During the same period, the U.S. Food and Drug Administration had regulations of several phthalates (e.g., DBP, BBP and DEHP) which can be used as the component of the food contact substances (e.g., adhesives, paper, and paperboard components, polymers, adjuvants like plasticizers) as indirect additives (21 CFR 175 to 178) (45). Thus, the production of phthalates and use/process of phthalates for the food related products may be different from the United States. Different regulations of phthalates in food contact substances in Taiwan and the USA could be one of the reasons for the discrepancies in NAHSIT (1993–1996 and 2005–2008) and NHANES (multiple survey cycles).

We determined that high unprocessed food consumption was associated with decreased levels of urinary phthalate metabolites, especially for MEP and MBP. Studies have reported negative associations between minimally processed food and urinary concentrations of HMW phthalate metabolites in the NHANES 2013–2014 and 2009–2016 (24, 25). One Korean intervention study reported that the urinary concentrations of MEP, MBP, and DEHP metabolites decreased after adherence to a strict vegetarian diet (plant-based) for 5 consecutive days (46). Overall, these studies suggested that unprocessed or minimally processed food consumption may lower the levels of phthalate exposure.

Our study determined that high ultra-processed intake was related to high LMW phthalate exposure. LMW phthalates can contaminate food through the following pathways: (1) use as a solvent and food packaging in the form of cardboard boxes with plasticized cellulose acetate windows and (2) use as components of printing ink and regenerated cellulose film coated with plasticized nitrocellulose to provide flexibility and heat retention (8, 20). Consequently, these LMW phthalates can migrate through volatilization or leach from food packaging into food; thus, high ultra-processed food intake could increase urinary levels of phthalate exposure.

A Swiss study demonstrated that a diet pattern based on “fatty, sweet, and ready meals” compared with a “healthy and natural” diet is associated with higher levels of DEHP and DBP exposure (47). The NHANES 2003–2008 reported that meat and energy intake were positively correlated with HMW metabolite and DEHP concentrations, but vegetable intake was negatively associated with LMW metabolites (48). Korean studies have also reported that a vegetarian diet can reduce DEP, DBP, and DEHP exposure (46). The food categories of meat, and dairy products contain higher concentrations of plasticizers than do cereals, vegetables, and fruits (9), which may be attributable to the ease of plasticizer deposition in fat-rich foods. Studies have indicated that high-fat dairy products such as cheese and butter contain high concentrations of plasticizers, and processed grain products such as bread are the primary sources of exposure for Belgians (49). These food categories may be related to the degree of food processing (9, 20).

Fat and sugar are often added to ultra-processed foods during processing to improve food palatability. These ingredients are generally added in grains, meat, or dairy products rather than vegetables and fruits. Furthermore, the possibility of exposure to plasticizers such as plastic packaging, gloves, and food additives may increase during processing (8). These phthalates may migrate and leach to food through food contact materials during preparation, storing and transportation. Even though plastics and related products are the main raw food packaging used in Taiwan, raw food are often washed, peeled, and heated before cooking, potentially reducing the levels of plasticizer exposure.

In this study, we found that those with a high level of education tend to consume more ultra-processed food; this result may be confounded by age because young adults tend to have a higher education level than older adults. In addition, our study demonstrated that ultra-processed food intake is increasing based on the NAHSIT II. This pattern was also observed in Taiwanese adolescents (27). Although the percentages of ultra-processed food intake in survey year 1993–1996 (NAHSIT I) were different as compared with those in survey year 2005–2008 (NAHSIT II), the patterns of these associations between urinary phthalate metabolites (such as MEP and MBP) and ultra-processed food intake were similar in two surveys (Supplementary Table S4). Furthermore, no significant interactions (*P* > 0.05) were found between quartiles of unprocessed/ultra-processed weight intake and survey year except for MBzP with ultra-processed food (*P* for interaction = 0.036) (data not shown).

To our knowledge, this is the first study to examine the level of food processing and its association with urinary phthalate metabolite exposure in an Asian population. Our study has several strengths. First, study participants were recruited from two waves of a representative nutrition dataset. The concentrations of phthalate metabolites and dietary intake reflect trends in a Taiwanese population. Second, we used 24-h dietary recall to collect comprehensive dietary data, which enabled us to classify the level of food processing. Furthermore, the half-life of phthalates is 12–48 h (50). The dietary information collected within the 24 h prior to an interview can better represent dietary exposure compared with food frequency questions measuring long-term exposure.

However, certain limitations must be noted. Firstly, this study employed a cross-sectional study design and may not have interpreted the causal relationship between diet and phthalates. Secondly, some residual confounders were not considered in this study (e.g., use of personal care products). Previous studies have detected different levels of DEP in personal care products such as face cream, shampoo and lotion (51). Thus, urinary levels of MEP could also be related to the use of personal care products (52). Previous studies indicated that urinary MEP levels in female were higher than in male because female used personal care products more frequently than males (53). However, no differences in urinary MEP levels were observed between female and male in the present study. Furthermore, no studies to date have reported associations between use of personal care products and unprocessed/ultra-processed food intake. Hence, we speculate that the influence of personal care product usage on our result could only be limited. Thirdly, we cannot rule out that social desirability may lead to the underestimation of ultra-processed or overestimation of unprocessed food intake which could dilute the association. Fourthly, the fact that dietary data did not capture all information indicative of food processing hampered the posterior application of NOVA classification and may lead to misclassification of exposure. Fifthly, the unweighted data used in the present study could not estimate the precise parameters from the whole population. However, a previous study reported similarity between weighted and unweighted analyses (54). Finally, we excluded half of participants from analysis due to ineligible or incomplete data. Thus, the finding of this study may not be generalized to the overall population.

In summary, our results from a sample of the general Taiwanese population indicated that ultra-processed food intake was associated with increased concentration of urinary MEP and that consuming unprocessed food was associated with lower concentrations of urinary MEP and MBP. Future large-scaled studies should confirm these findings and explore the mechanisms of action.

Our study findings have several policy implications. Public health policies and market incentives should be developed to promote the consumption of unprocessed foods (and freshly prepared meals) and minimize that of ultra-processed foods. Furthermore, public awareness and education are needed about the possible risk of eating ultra-processed food, and advocate on environmentally friendly packaging into food system. The government needs to monitor the general population in order to assess the impacts of ultra-processed food intake and phthalate exposure on health.

## Data Availability Statement

The original contributions presented in the study are included in the article/Supplementary Material, further inquiries can be directed to the corresponding authors.

## Ethics Statement

The studies involving human participants were reviewed and approved by the Research Ethics Committee of China Medical University and Hospital (No: CMUH108-REC2-174). The patients/participants provided their written informed consent to participate in this study.

## Author Contributions

Y-CH, P-RH, and H-BH: conceptualization. Y-CH and H-BH: methodology. S-LW: investigation and data curation. Y-CH and P-RH: formal analysis. C-WS: experimental analysis. Y-CH and Y-TL: writing—original draft. Y-CH, Y-TL, S-LW, and H-BH: writing—review and editing. W-HP: critical revision of the manuscript. S-LW and H-BH: supervision. All authors contributed to the article and approved the submitted version.

## Funding

This work was supported by China Medical University (Grant number: CMU108-N-16); and the Ministry of Science and Technology (Grant numbers: MOST 109-2314-B-016-025 and MOST 110-2628-B-016-003). The funders had no role in the study design; in the collection, analysis and interpretation of data; in the writing of the report; or in the decision to submit the article for publication.

## Conflict of Interest

The authors declare that the research was conducted in the absence of any commercial or financial relationships that could be construed as a potential conflict of interest.

## Publisher's Note

All claims expressed in this article are solely those of the authors and do not necessarily represent those of their affiliated organizations, or those of the publisher, the editors and the reviewers. Any product that may be evaluated in this article, or claim that may be made by its manufacturer, is not guaranteed or endorsed by the publisher.
