# Supplementary material for: Food Processing and Phthalate Exposure: The Nutrition and Health Survey in Taiwan (1993–1996 and 2005–2008)
Source: Front Nutr. 2021 Nov 17;8:766992. doi: 10.3389/fnut.2021.766992 (PMC8635797; doi:10.3389/fnut.2021.766992)
Supplement: Supplementary file 1 [file Table_1.docx]

Supplementary Material

### Supplementary Table S1. Examples foods by unprocessed and ultra-processed foods

| Food group | Examples |
| --- | --- |
| Unprocessed or minimally processed foods | 1. Meat (includes poultry) and viscera 2. Fruit and 100% fruit juices 3. Milk (pasteurized, fermented or powdered) and plain yogurt 4. Whole grains: sticky rice, rice ball, porridge, corn, red beans 5. Eggs 6. Legumes 7. Fish and seafood 8. Vegetables 9. Others: nuts, avocado |
| Ultra-processed foods | 1. Breads: Taiwanese bread, toast, bagels, croissant, French bread, focaccia, hot dog/hamburger bun 2. Drinks: soft drinks, not 100% juice, soda, sweetened/artificially tea, soy milk, rice milk, coffee, flavor drink powder 3. Cookies: packaged crackers, cookies, pudding, chocolate, mints, candy, fudge, tongue cake, nougat, puffs, mochi, jelly 4. Frozen and ready-to-eat product: frozen Chinese Dim Sum (e.g. dumplings, streamed bun), ready-to-eat pizza, instant noodle, convenience store onigiri, pre-prepared or packaged pies 5. Grain products: flavor breakfast cereals, corn flake, instant oatmeal, instant cereal powder 6. Sauces and dressings: shacha sauce, douban sauce, commercially-made broth, salad dressing, barbecue sauce, instant soup 7. Reconstituted meat: meat ball, fish ball, hot dog, chicken nuggets, sausage 8. Milk-based products: flavor milk, flavor yogurt, ice cream, bar, stick 9. Sweet snacks: cakes, pineapple cake, suncake, moon cake 10. Fried foods: French fries, potato chips, sweet potato chips 11. Others: margarine, vegetarian meat substitutes |

**Supplementary Table S2.** MeansSD of food weight and calorie contribution by the four NOVA groups

|  | Unprocessed or minimally processed foods | Processed culinary ingredients | Processed foods | Ultra-processed foods |
| --- | --- | --- | --- | --- |
| Weight, % | 71.321.4 | 3.855.54 | 8.6012.2 | 16.318.6 |
| Calorie, % | 56.322.9 | 12.511.6 | 12.514.7 | 18.819.7 |

**Supplementary Table S3.** Percentage difference (95% confidence interval) in urinary phthalate metabolites (μg/L) by quartiles of unprocessed and ultra-processed food intake (*n* = 516)

| – | | | Quartiles | | | | *P* for trend |
| --- | --- | --- | --- | --- | --- | --- | --- |
|  |  |  | 1 | 2 | 3 | 4 |  |
|  |  |  | Unprocessed food | | | |  |
|  | MMP |  | 0 | 3.35 (-26.1, 44.4) | -3.31 (-31.7, 36.8) | 0.09 (-30.5, 44.2) | 0.91 |
|  | MEP |  | 0 | 10.7 (-27.4, 68.9) | -8.21 (-40.8, 42.2) | -37.9 (-60.8, -1.58) | **0.03** |
|  | MBP |  | 0 | -9.22 (-26.1, 11.6) | -10.4 (-27.6, 11.0) | **-23.1 (-38.6, -3.71)** | **0.02** |
|  | MBzP |  | 0 | -3.04 (-28.9, 32.2) | 7.20 (-22.2, 47.8) | 7.52 (-23.3, 50.8) | 0.55 |
|  | ΣDEHP (μmol/L) |  | 0 | 6.07 (-16.3, 34.5) | 12.8 (-11.8, 44.2) | -16.6 (-35.6, 8.04) | 0.26 |
|  |  |  | Ultra-processed food | | | |  |
|  | MMP |  | 0 | 6.76 (-24.1, 50.1) | 0.18 (-33.8, 41.5) | 3.56 (-27.9, 48.8) | 0.93 |
|  | MEP |  | 0 | **54.2 (0.37-137)** | **75.2 (13.3, 171)** | **63.8 (3.66, 159)** | **0.02** |
|  | MBP |  | 0 | 12.2 (-9.05, 38.4) | 22.3 (-1.19, 51.3) | 10.6 (-11.6, 38.3) | 0.27 |
|  | MBzP |  | 0 | -0.62 (-27.5, 36.2) | 13.2 (-17.8, 55.7) | -1.87 (-29.9, 37.3) | 0.89 |
|  | ΣDEHP (μmol/L) |  | 0 | 4.50 (-18.0, 33.2) | 0.00 (-21.8, 27.9) | -0.07 (-21.8, 27.9) | 0.92 |

Model was adjusted for survey year, age, gender, body mass index, education level, urinary creatinine, total energy intake, and total fat intake.

*P* < 0.05 is presented in bold.

**Supplementary Table S4.** Percentage difference (95% confidence interval) in urinary phthalate metabolites (μg/L) by quartiles of unprocessed and ultra-processed food intake by two surveys

| Variables | | | Quartiles | | | | *P* for trend |
| --- | --- | --- | --- | --- | --- | --- | --- |
|  |  |  | 1 (Low) | 2 | 3 | 4 (High) |  |
|  |  |  |  | | | |  |
| NAHSIT 1993-1996  (*n* = 197) | Unprocessed food | |  |  |  |  |  |
|  | MMP |  | 0 | 2.54 (-23.5, 54.2) | 27.8 (-15.5, 93.2) | -12.3 (-43.0, 34.9) | 0.782 |
|  | MEP |  | 0 | -3.99 (-46.6, 72.7) | -12.1 (-51.5, 59.4) | **-53.0 (-74.7, -12.7)** | **0.015** |
|  | MBP |  | 0 | -21.0 (-45.5, 14.6) | -11.8 (-39.5, 28.6) | **-35.7 (-56.5, -4.76)** | 0.061 |
|  | MBzP |  | 0 | 12.6 (-33.1, 89.4) | **69.5 (0.05, 187)** | -14.3 (-50.5, 45.3) | 0.908 |
|  | ΣDEHP (μmol/L) |  | 0 | 24.3 (-21.8, 97.5) | 22.8 (-23.2, 96.4) | -11.0 (-45.4, 45.1) | 0.572 |
|  | Ultra-processed food | |  | | | |  |
|  | MMP |  | 0 | 9.17 (-24.7, 58.3) | 40.3 (-6.16, 110) | 20.0 (-22.3, 84.8) | 0.219 |
|  | MEP |  | 0 | 32.9 (-22.6, 128) | 55.4 (-13.5, 179) | 51.4 (-19.3, 184) | 0.137 |
|  | MBP |  | 0 | 25.8 (-10.5, 76.6) | 31.3 (-9.11, 87.7) | 11.4 (-25.0, 65.5) | 0.425 |
|  | MBzP |  | 0 | 8.37 (-31.9, 76.0) | **71.4 (2.52, 186)** | -7.72 (-46.9, 60.3) | 0.653 |
|  | ΣDEHP (μmol/L) |  | 0 | -20.2 (-47.6, 21.8) | -20.4 (-49.3, 25.8) | -14.1 (-47.4, 40.4) | 0.470 |
|  |  |  |  |  |  |  |  |
| NAHSIT 2005-2008  (*n* = 319) | Unprocessed food |  | 0 |  |  |  |  |
|  | MMP |  | 0 | 1.74 (-37.0, 64.3) | -21.3 (-52.4, 30.2) | 6.49 (-37.9, 82.7) | 0.900 |
|  | MEP |  | 0 | 16.8 (-34.7, 109) | -12.3 (-52.3, 61.5) | -30.8 (-64.0, 33.3) | 0.217 |
|  | MBP |  | 0 | -2.73 (-23.4, 23.8) | -8.92 (-29.3, 17.3) | -16.2 (-36.1, 9.93) | 0.184 |
|  | MBzP |  | 0 | -10.2 (-38.5, 31.3) | -20.2 (-46.3, 18.8) | 22.0 (-20.4, 87.0) | 0.575 |
|  | ΣDEHP (μmol/L) |  | 0 | -3.84 (25.7, 24.5) | 7.05 (-18.3, 40.3) | -19.4 (-36.7, 7.82) | 0.292 |
|  | Ultra-processed food |  |  |  |  |  |  |
|  | MMP |  | 0 | -1.58 (-42.6, 68.6) | -22.7 (-54.2, 30.6) | -5.11 (-44.4, 61.9) | 0.685 |
|  | MEP |  | 0 | 83.7 (-4.10, 252) | **108 (10.3, 291)** | 83.6 (-3.64, 250) | 0.082 |
|  | MBP |  | 0 | 2.61 (-21.8, 34.6) | 11.4 (-14.5, 45.0) | 9.93 (-16.0, 43.9) | 0.413 |
|  | MBzP |  | 0 | -14.2 (-44.1, 31.6) | -14.4 (-43.6, 29.8) | -4.43 (-37.5, -46.1) | 0.888 |
|  | ΣDEHP (μmol/L) |  | 0 | 29.5 (-3.11, 73.1) | 16.8 (-12.0, 54.9) | 13.7 (-14.7, 51.6) | 0.604 |

Model was adjusted for age, gender, body mass index, education level, total energy intake, and urinary creatinine.

*P* < 0.05 is presented in bold.
